# Dataset from RNAseq analysis of differential gene expression among developmental stages of two non-marine ostracodes

**DOI:** 10.1016/j.dib.2024.110070

**Published:** 2024-01-17

**Authors:** Miguel Vences, Sten Anslan, Joana Sabino-Pinto, Mauricio Bonilla-Flores, Paula Echeverría-Galindo, Uwe John, Benneth Nass, Liseth Pérez, Michaela Preick, Liping Zhu, Antje Schwalb

**Affiliations:** a Zoological Institute, Technische Universität Braunschweig, Mendelssohnstr. 4, 38106 Braunschweig, Germany; b Institute of Ecology and Earth Sciences, University of Tartu, Juhan Liivi 2, 50409 Tartu, Estonia; c Department of Biological and Environmental Science, University of Jyväskylä, Jyväskylä, Finland; d Groningen Institute for Evolutionary Life Sciences, University of Groningen, Nijenborgh 7, 9747 AG Groningen, the Netherlands; e Institute of Geosystems and Bioindication, Technische Universität Braunschweig, Langer Kamp 19c, 38106 Braunschweig, Germany; f Alfred-Wegener-Institut Helmholtz-Zentrum für Polar- und Meeresforschung, Am Handelshafen 12, 27570 Bremerhaven, Germany; g Faculty of Mathematics and Natural Sciences, Institute for Biochemistry and Biology, University of Potsdam, Karl-Liebknecht-Str. 24-25, 14476 Potsdam, Germany; h Institute of Tibetan Plateau Research, Chinese Academy of Sciences, 16 Lincui Road, Beijing 100101, China

**Keywords:** Ostracoda, *Dolerocypris*, *Heterocypris*, Transcriptomics, Phylogenomics

## Abstract

We contribute transcriptomic data for two species of Ostracoda, an early-diverged group of small-sized pancrustaceans. Data include new reference transcriptomes for two asexual non-marine species (*Dolerocypris sinensis* and *Heterocypris* aff. *salina*), as well as single-specimen transcriptomic data that served to analyse gene expression across four developmental stages in *D. sinensis.* Data are evaluated by computing gene expression profiles of the different developmental stages which consistently placed eggs and small larvae (at the stage of instar A-8) similar to each other, and apart from adults which were distinct from all other developmental stages but closest to large larvae (instar A-4). We further evaluated the transcriptomic data with two newly sequenced low-coverage genomes of the target species. The new data thus document the feasibility of obtaining reliable transcriptomic data from single specimens – even eggs – of these small metazoans.

Specifications Table

**Table:** 

Subject	Biological sciences / Omics: Transcriptomics
Specific subject area	Descriptive work on the transcriptome of two marine ostracodes as well as age-related differences in gene expression.
Data format	Raw, Analyzed
Type of data	Tables, figures
Data collection	The data was sequenced from specimens of *Dolerocypris sinensis* and *Heterocypris* aff. *salina*. RNA was extracted using a standard trizol protocol, the DNA with QIAGEN MagAttract HMW DNA Kit. Sequencing was performed with Illumina NextSeq 500/550. Transcriptomes were processed with: VSEARCH v2.15.0, DIAMOND v2.0.6, Trinity v2.11.0, rnaQUAST v2.2.0. Genomes were processed with the Supernova pipeline v 2.1.1.
Data source location	Specimens of *Dolerocypris sinensis* were collected in June 2019 from an exhibition container with abundant aquatic vegetation at the Botanical Garden of the Technische Universität Braunschweig, Germany (geographical coordinates 52.27083, 10.53306). Specimens of *Heterocypris* aff. *salina* were collected in September 2019 from an ephemeral pond (30.78911, 90.96406) next to Nam Co, Tibet, China.
Data accessibility	Repository name: NCBI Sequence Read Archive (SRA) Data identification number: BioProject ID PRJNA972629 BioSamples: SAMN35343723, SAMN35343722 (reference transcriptome data); SAMN35564813–SAMN35564832 (single-individual RNAseq data); SAMN35084548 and SAMN35084547 (draft genome data). Direct URL to data: https://www.ncbi.nlm.nih.gov/bioproject/?term=PRJNA972629 Repository Name: Zenodo Data identification number: 10.5281/zenodo.7704680 Direct URL to data: https://doi.org/10.5281/zenodo.7704680

## 1 Value of the Data

• The data are useful as they contribute genomic resources to an ancient group of arthropods of importance as environmental indicators [[1], [2], [3], [4]] and with a very low number of sequenced genomes and transcriptomes [[5], [6], [7], [8]].
• The data will be of interest for researchers focusing on comparative arthropod genomics and phylogenetics, and evolutionary developmental researchers focusing on the larval development of ecdysozoan animals.
• The data can be re-used as a source for sequences in future phylotranscriptomic analysis of ostracode evolutionary relationships, and for characterization of phylogenetic markers for this group of crustaceans. They can also be directly used in meta-analyses of genes involved in arthropod and ecdysozoan larval development.

## 2 Objective

We aim to contribute to the availability of genomic resources of ostracodes, specifically for the two asexual species, *Dolerocypris sinensis* from Germany and *Heterocypris* aff. *salina* from China. In small organisms such as ostracodes (body lengths < 2 mm and sometimes as small as 0.2 mm), it is usually necessary to pool multiple individuals to obtain the necessary quantities of nucleic acids for sequencing purposes. We instead used primarily a RNAseq approach usually applied to single-cell transcriptomics to characterize gene expression of single individuals of different developmental stages of one of our focal species. We also present high-coverage reference transcriptomes and low-coverage draft genome data for these two species.

## 3 Data Description

The dataset deposited in the NCBI Sequence Read Archive (SRA) under BioProject ID PRJNA972629 includes all raw sequence data (https://www.ncbi.nlm.nih.gov/bioproject/?term=PRJNA972629).

**Fig. 1:**
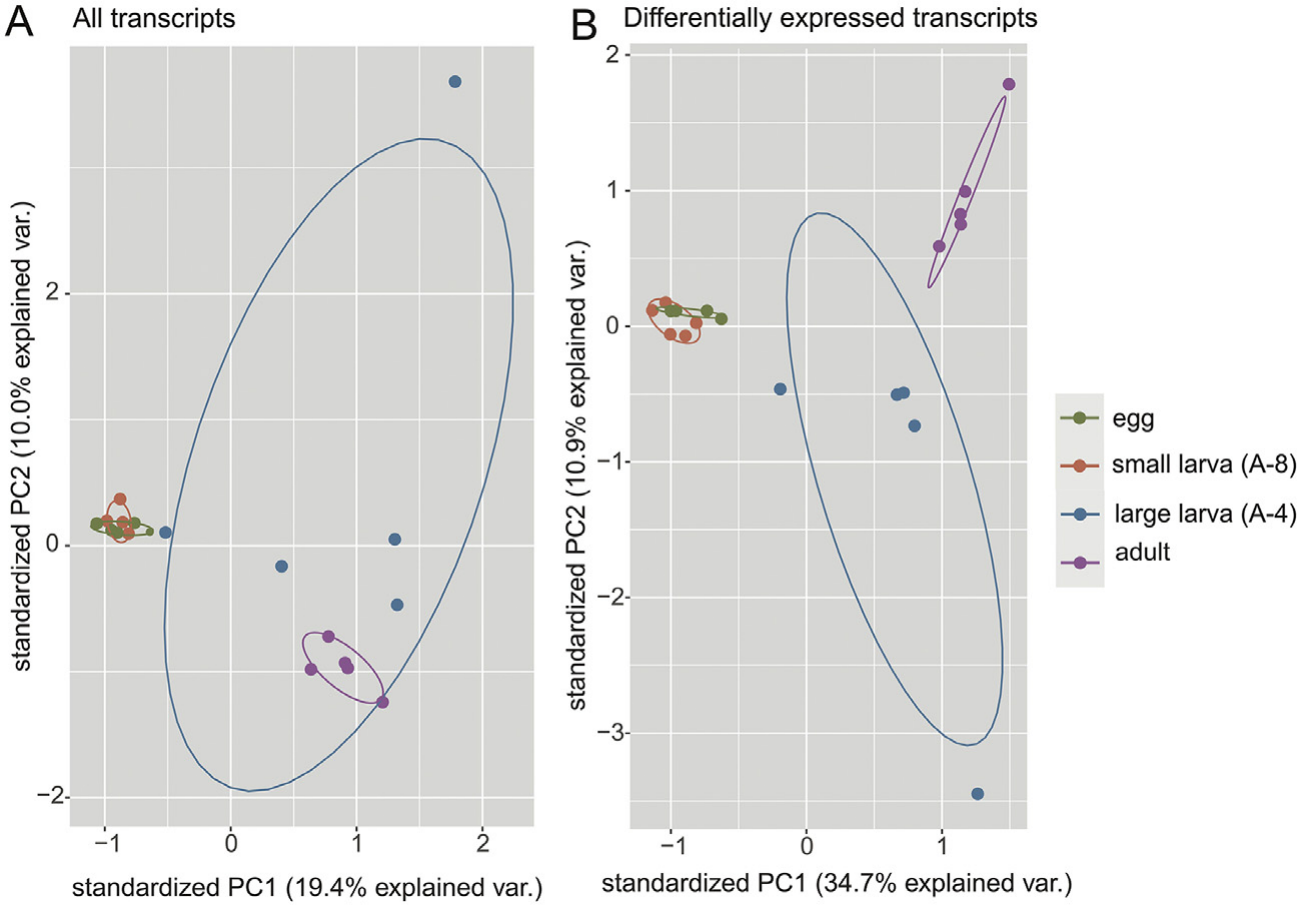
Scatterplots of Principal Components 1 and 2 (PC1, PC2) from Principal Component Analyses (PCAs) of gene expression profiles corresponding to *Dolerocypris* individuals in different developmental stages (large larva, instar A-4 and small larva, instar A-8), calculated from (A) all transcripts and (B) only the transcripts found to be differentially expressed transcripts. The plots validate the individual-ostracode RNAseq data by demonstrating coherent clustering by developmental stage.
(3) Low-coverage genomes for each of the target species: BioSamples SAMN35084548 and SAMN35084547.

(1) Reference transcriptomes for the two target species: BioSamples SAMN35343723 and SAMN35343722.
(2) Single-individual RNAseq data for 20 specimens in four developmental stages: BioSamples SAMN35564813- SAMN35564832. Fig. 1 illustrates the gene expression profiles corresponding to *Dolerocypris sinensis* individuals in different developmental stages. A list of *D. sinensis* individuals used for the single-ostracode RNAseq analyses noting the instar stage, lab details and number of obtained reads is provided in Table 2.

The Zenodo repository (DOI 10.5281/zenodo.7704680) furthermore contains genome and transcriptome assembly and annotation files, as well as data used for gene expression analysis and phylogenetic validation of selected key markers. The summary statistics describing the reference transcriptome assemblies are outlined in Table 1. Supplementary Table S1 (also available from the Zenodo repository) provides a full list of GenBank accession numbers of sequences used for decontamination procedures of ostracode genome and transcriptome data sets. Supplementary Table S2 (also available from the Zenodo repository) provides a list of differentially expressed genes across developmental stages of *D. sinensis.*

**Table 1:** Summary statistics for the assemblies of the reference transcriptomes.

Variable	*Dolerocypris sinensis*	*Heterocypris* aff. *salina*
Minimum length (bp)	187	190
Maximum length (bp)	19,205	22,552
Mean length (bp)	990	1184
N50 (bp)	2098	2303
Number of transcripts	164,065	190,657
Number of transcripts > 1kb	47,171	70,586
Number of bases	1.62E+08	2.26E+08
GC content	0.4927	0.4793

Expression profiles of individual organisms are of importance as they allow, for instance, a better understanding of idiosyncratic responses of individuals to environmental factors which may be blurred when analyzing pooled samples. Our data demonstrate that such approaches can be successfully applied to individual ostracodes. On average across all 20 individual transcriptomes, mean transcript length was 1383 bp, N50 was 2567 bp, and GC content was 0.524. As revealed by Principal Component Analyses, the single-ostracode transcriptome data reflect the expected differences between developmental stages. Specifically, the expression profiles of all eggs and all A-8 instar larvae were very similar to each other (Fig. 1; see Supplementary Table S2 for genes overexpressed per stage and their molecular functions). The larger larvae (instar A-4) had a higher in-group variation. Adults were distinct from all other groups, but closest to large larvae in the PCA plots (Fig. 1). Egg samples had overexpressed transcripts with gene ontology terms related to cell replication such as *mitotic recombination-dependent replication fork processing* and *cell division*. Young larvae (instar A-8) had overexpressed transcripts related to various cell biological processes while for older larvae (instar A-4), among the overexpressed transcripts it is worth mentioning two genes related to development, such as *regulation of cell shape* and *chitin catabolic process*. Adults had overexpressed transcripts related to responses to stress such as *response to oxidative stress* and *defense response to bacterium*. Of the top-10 overexpressed genes in adults, nine were exclusively represented in this stage, with no reads recovered in any other stage. See Table S2 in the Zenodo repository referred to on “Data description” section for a detailed overview of gene ontologies of differentially expressed transcripts.

## 4 Experimental Design, Materials and Methods

### 4.1 Experimental design

Transcriptome comparisons were focused on eggs, larvae in stages A-8 and A-4, and adults. Specimens of *Dolerocypris sinensis* were collected in June 2019 from an exhibition container with abundant aquatic vegetation at the Botanical Garden of the Technische Universität Braunschweig, Germany (geographical coordinates 52.27083, 10.53306). Specimens of *Heterocypris* aff. *salina* were collected in September 2019 from an ephemeral pond (30.78911, 90.96406) next to Nam Co, Tibet, China. They were stored in pond water in a 1 l transparent plastic bottle, transported to the respective laboratory, and individually bred for a period of approximately three months in transparent six-well plates (each well 15 ml) and fed with spinach. For transcriptome sequencing, we selected matrilines with sufficient specimens (30–40 adults or larvae in the stage of instar A-1, all from one matriline) without visual signs of fungal or bacterial contamination.

### 4.2 RNA extraction and RNAseq

For sequencing reference transcriptomes, samples of 30 specimens of *D. sinensis* and 40 specimens of *H.* aff. *salina* (mixed larval stages and matrilines) were pooled according to species, smashed with a micropestle, and stored in separate Eppendorf vials containing RNAlater. Vials were incubated for 1 h at room temperature and were subsequently frozen at −20 °C and finally at −80 °C. RNA was extracted using a standard trizol protocol. Libraries were prepared and sequenced on an Illumina NextSeq instrument using a High Output v2 150 cycle kit.

For RNAseq of individual ostracodes, five ostracodes per developmental stage were selected. Each specimen was smashed with a micropestle and each stored individually in a PCR tube with 100 ml of lysis buffer. Vials were incubated for 1 h at room temperature and were subsequently frozen at −20 °C and finally at −80 °C. RNA from the individual ostracode specimens and eggs was extracted using an RNAqueous®-Micro Kit (Invitrogen) following manufacturer's instructions. RNA samples were stored at −70 °C until complementary DNA (cDNA) synthesis with a SMART-Seq® v4 Ultra® Low Input RNA Kit (Takara Bio USA, Inc.) following the manufacturer's instructions (PCRs with 10–18 cycles). Amplified cDNA was purified using the CleanNGS magnetic bead kit (CleanNA) and eluted with 15 µl of Elution Buffer. Before sequencing, samples were uniquely indexed using Nextera XT DNA Library Prep Kit, following manufacturer's instructions. Samples were pooled in equimolar concentrations and the final concentration of the cDNA pool for sequencing was 2 nM. Sequencing was performed with Illumina NextSeq 500 (2 × 150 bp).

### 4.3 Assembly, quality filtering and removal of non-target reads from RNAseq data

Raw Illumina reads of reference transcriptomes were quality-filtered using VSEARCH v2.15.0 [9] by removing reads with expected error rate of >1 (–fastq_maxee 1) and reads containing ambiguous bases (–fastq_maxns 0). Before assembly, we performed DIAMOND blastx searches (v2.0.6 [10]) against custom bacterial, fungal, virus, plant, invertebrate, and protist databases (see Table S1 in the Zenodo repository referred to in the “Data description” section; data downloaded on 17.08.2020 from NCBI) to remove potential non-target sequences. We performed a ‘sensitive’ search (–sensitive) and excluded contigs that got hit against the custom databases outlined above with the e-value threshold of 1E-16. Average sequencing depth (number of sequences) per sample was 226,979,459; after raw data filtering steps (as described above), the average sequence count per sample was 153,929,721. Filtered RNAseq reads were *de novo* assembled with Trinity (v2.11.0) using default parameters [11]. Assembly quality evaluation was performed with rnaQUAST (v2.2.0 [12]). A total of 164,065 and 190,657 transcripts were generated with a N50 of 2098 and 2303 for *D. sinensis* and *H.* aff. *salina*, respectively; assembly summary statistics are detailed on Table 1. Transcripts were translated into proteins using TransDecoder on the Galaxy platform [13]. Assemblies were subsequently cleaned by removing potential non-target sequences in one additional DIAMOND search with the same settings as for genome assemblies.

For individual ostracode RNAseq, the raw Illumina sequences were quality filtered using VSEARCH by trimming out low quality (<5) regions and passing reads with maximum expected error rate of 0.5, with no ambiguous bases (–fastq_truncqual 5, –fastq_maxee 0.5, –fastq_maxns 0). To remove potential contaminant bacterial sequences, we performed DIAMOND blastx searches of the quality-filtered reads against a custom bacterial database as described above. Average sequencing depth per sample was 17,878,326; after raw data filtering steps, the average sequencing depth per sample was 10,331,325 (Table 2). The filtered RNAseq reads of the 20 *H.* aff. *salina* individuals were aligned to the reference transcriptome assembly described above with Bowtie (v1.1.2 [14]), and quality of the assemblies evaluated with rnaQUAST (v2.2.0 [12]).

**Table 2:** List of individuals of *Dolerocypris sinensis* used for single-ostracode RNAseq and gene expression analysis. All specimens originated from two matrilines (M1/M2) originally collected from the Botanical Garden in Braunschweig, Germany. *eggs with black dots; # all adults had visible eggs inside their body cavity.

Sample ID	matriline	larval stage (instar)	RNA conc. (ng/ul) for cDNA synt.	PCR cycles	Amount of DNA for Nextera lib prep	Biosample	Raw reads	Filtered reads
**1a**	M2	A-8	3.83	15	150 pg	SAMN35564813	19,238,790	11,032,997
**1b**	M2	A-8	3.43	18	150 pg	SAMN35564814	22,300,601	11,292,395
**1c**	M2	A-8	2.21	15	150 pg	SAMN35564815	16,543,768	9,879,280
**1d**	M1	A-8	2.62	18	150 pg	SAMN35564816	19,799,081	11,542,467
**1e**	M1	A-8	2.53	18	150 pg	SAMN35564817	20,500,480	11,463,711
**2a**	M1	egg	2.14	18	150 pg	SAMN35564818	16,770,977	10,103,781
**2b**	M1	egg	3.40	18	150 pg	SAMN35564819	24,035,698	13,171,607
**2c**	M1	egg	2.79	18	150 pg	SAMN35564820	19,632,988	9,574,757
**2d**	M2	egg *	2.17	18	150 pg	SAMN35564821	18,170,146	11,467,667
**2e**	M2	egg *	2.89	18	150 pg	SAMN35564822	13,405,023	7,598,702
**3a**	M1	A-4	2.15	12	150 pg	SAMN35564823	18,455,413	10,310,825
**3b**	M1	A-4	2.00	10	150 pg	SAMN35564824	16,069,512	10,208,787
**3c**	M1	A-4	1.95	14	150 pg	SAMN35564825	10,510,239	6,132,827
**3d**	M2	A-4	1.45	14	150 pg	SAMN35564826	17,876,801	9,818,283
**3e**	M2	A-4	2.46	14	150 pg	SAMN35564827	15,541,360	9,460,626
**4a**	M1	Adult #	2.40	10	150 pg	SAMN35564828	16,388,546	9,786,663
**4b**	M1	Adult #	2.60	12	150 pg	SAMN35564829	17,603,689	9,932,933
**4c**	M2	Adult #	2.10	12	150 pg	SAMN35564830	18,017,693	11,071,164
**4d**	M2	Adult #	2.10	14	150 pg	SAMN35564831	17,563,188	10,220,211
**4e**	M2	Adult #	2.00	10	150 pg	SAMN35564832	19,142,529	12,556,832

### 4.4 Genome sequencing

DNA was extracted from six specimens per species placed into 220 µl ATL buffer (Qiagen) and ground with a micropestle (Thermo Fisher, Z137314). The resulting suspension was further processed using the MagAttract HMW gDNA Kit (Qiagen) following the supplier's instructions. Elution was performed into 150 µl of AE buffer (Qiagen). DNA quantity and quality were checked using Tape Station HSD1000 Kit (Agilent) and Qubit (Thermo Fisher, Q32854). Using 0.99 ng of DNA for one sample (*H*. aff. *salina*) and 0.93 ng for the second sample (*D. sinensis*) (each sample from pooled individuals; see above), 10X Chromium sequencing libraries were prepared with the respective Genome Reagent Kit following the supplier's instructions. They were quantified using the NEBNext Library Quant Kit (New England Biolabs) on a PikoReal 96 Real-Time PCR machine (Thermo Fisher Scientific TCR0096). For sequencing we used 2.0 picomoles of each library, i.e., for *D. sinensis* (library concentration 4 nM) we used 2.23 µl and for *H*. aff. *salina* (library concentration 1.91 nM) we used 10.00 µl. Both samples were separately sequenced on an Illumina NextSeq 500/550 instrument in 150 bp PE mode, using a high output kit corresponding to approximately 400 million reads. *De novo* assemblies were performed with the Supernova pipeline (v2.1.1; 10X Genomics, 2020), following the manufacturer's instructions. Genome annotation was performed using MAKER v2.31.11, integrating evidence from the reference transcriptome assemblies, protein alignments, and functional annotation [15].

### 4.5 Gene expression analysis

Differences in transcript abundance were identified with Trinity using the edgeR (v3.20.9 [16]) method and default parameter values. Abundance differences were determined between each life stage and all the others combined (e.g., eggs vs. instar A-8 + instar A-4 + adults) and between younger and older stages (eggs + instar A-8 vs. instar A-4 + adults). Differentially expressed transcripts were annotated with Trinotate (v3.1.1) using default parameter values [17]. Principal Component Analyses (PCAs) were computed based on transcript abundances, both for all transcripts and for all differentially expressed transcripts, on R (v4.0.0 [18]) with the package ggbiplot [19]. Gene ontology terms for differentially expressed genes were determined with QuickGO [20].

## Limitations

The draft genome sequences obtained were highly incomplete, with Arthropoda-BUSCO scores of 44.4 % (*D. sinensis*) and 73.2 % (*H.* aff. *salina*) for the genome annotation. This pattern was exacerbated by our rigorous decontamination procedures which, however, ensured a high accuracy of the remaining annotated genes which therefore served to validate the reference transcriptomes in exploratory phylogenetic analyses of universal arthropod single-copy marker genes from [21]. The genomes were however not used further analysed due to their incompleteness (24,736 and 24,216 scaffolds for *D. sinensis* and *H.* aff. *salina*, respectively, with maximum scaffold lengths of 2349,980 and 2371,011 bp and N50 of 38,868 and 21,055 bp; 12,485 and 20,733 gene models with average lengths of 3506 and 3108 bp after annotation). Further details of the genome annotation and alignments of these genes are available from the Zenodo repository.

## Ethics Statement

Permits for the study in China were obtained via the Institute of Tibetan Plateau Research (Chinese Academy of Sciences) from the Tibet Autonomous Region Government (issued on 19 June 2018). Breeding and sacrificing ostracodes does not require specific ethics approval according to German, Chinese or European law (EU Directive 2010/63/EU for animal experiments) but was nevertheless carried out in ways to minimize any stress or suffering of these animals.

## CRediT authorship contribution statement

**Miguel Vences:** Conceptualization, Data curation, Supervision, Writing – original draft, Writing – review & editing. **Sten Anslan:** Conceptualization, Data curation, Investigation, Formal analysis, Writing – original draft, Writing – review & editing. **Joana Sabino-Pinto:** Formal analysis, Writing – original draft, Writing – review & editing. **Mauricio Bonilla-Flores:** Investigation, Formal analysis, Resources, Writing – review & editing. **Paula Echeverría-Galindo:** Investigation, Resources, Writing – review & editing. **Uwe John:** Methodology, Conceptualization, Writing – review & editing. **Benneth Nass:** Formal analysis, Writing – review & editing. **Liseth Pérez:** Conceptualization, Investigation, Writing – review & editing. **Michaela Preick:** Investigation, Methodology, Writing – review & editing. **Liping Zhu:** Resources, Supervision, Writing – review & editing. **Antje Schwalb:** Supervision, Writing – review & editing.

## Data Availability

Bioproject PRJNA972629 (Original data) (NCBI) Dataset from RNAseq analysis of differential gene expression among developmental stages of two non-marine ostracods (Original data) (Zenodo)

## Appendix Supplementary materials

Image, application 1

Image, application 2
